# Is Greed a Double-Edged Sword? The Roles of the Need for Social Status and Perceived Distributive Justice in the Relationship Between Greed and Job Performance

**DOI:** 10.3389/fpsyg.2019.02021

**Published:** 2019-08-28

**Authors:** Yiming Zhu, Xiaomin Sun, Sijia Liu, Gang Xue

**Affiliations:** ^1^Beijing Key Laboratory of Applied Experimental Psychology, National Demonstration Center for Experimental Psychology Education (Beijing Normal University), Faculty of Psychology, Beijing Normal University, Beijing, China; ^2^Department of Public Administration, Chinese Academy of Governance, Beijing, China

**Keywords:** greed, perceived distributive justice, need for social status, task performance, contextual performance

## Abstract

Greed is one of the most common features of human nature, and it has recently attracted increasing research interest. The aims of this paper are to provide one of the first empirical investigations of the effects of greed on job performance and to explore the mediating role of the need for social status and perceived distributive justice. Using a working sample (*N* = 315) from China, the current study found that greed promoted both task and contextual performance through the intermediary effect of the need for social status. At the same time, greed inhibited both types of performance through perceived distributive justice. These results confirmed our hypothesis that greed is a double-edged sword with opposite effects on one’s performance. The findings suggest that organizations should both address greedy employees’ social status concerns and ensure that they are treated fairly so that organizations can fully utilize the talents of greedy people and channel their energy in a beneficial direction.

## Introduction

The discussion on greed is as old as the discussion on wealth and power. Although a consensus has been reached that greed is a common and inevitable part of human nature (Balot, 2001; Wang et al., 2011), people seem to hold different attitudes toward greed. The famous quote by the lead character in the movie *Wall Street* says, “greed… captures the essence of the evolutionary spirit. Greed…has marked the upward surge of mankind.” In fact, greed is highly valued in some organizations and societies (Bruhn and Lowrey, 2012). In contrast, almost all religions treat greed as immoral and evil (Wang et al., 2011). Similarly, studies have suggested that greed is associated with many negative characteristics (Gilliland and Anderson, 2011). It makes people focus only on their personal fulfillment and satisfaction, ignoring norms and values (Levine, 2005), and this focus may explain why greed is associated with such negative behaviors as fraud (Smith, 2003), deception (Cohen et al., 2009), theft (Caudill, 1988), corruption (Rose-Ackerman, 1999), and other unethical behaviors (Seuntjens et al., 2019).

Many economists tend to believe that greed is good, claiming that greed is the driving force of economic development (Greenfeld, 2003). The argument is that if people are eager to maximize their own interests and are never satisfied with their current possessions, they will eventually engage in activities that are beneficial to the whole society (Oka and Kuijt, 2014). The underlying logic of this proposition is that greed promotes individual performance, which in turn benefits the development of organizations and societies. However, there is a missing link regarding whether greed facilitates individual performance.

The relationship between greed and performance is far from self-evident. In organizational settings, where individuals work together with others and social status is valued even more highly than money (Ezard, 2000). Thus, employees with higher level of greed are more likely to have a higher level of the need for social status, which makes them work harder to gain the status they long for. Meanwhile, greed may distort people’s perception of distributive justice, which may in turn hold them back from devoting themselves to their work. In sum, it seems two different paths may exist through which greed could affect performance. In the current study, we tested the facilitating effect of greed on performance through the need for social status and the inhibiting effect of greed on performance through perceived distributive justice.

Greed is an inherent part of human nature, and performance prediction is of great importance for organizational research and practice. Organizational psychologists Bruhn and Lowrey (2012) claimed that greed could simultaneously have both positive and negative impacts on organizational and individual performance, but until now, few empirical studies have tested whether and how greed promotes or diminishes employees’ performance. The current study aimed to depict the nuanced relation and inner mechanisms between greed and performance. The results are helpful for researchers and practitioners to reflect under what conditions could greed improve or decrease performance, which is crucial from both academic and practical viewpoints.

### Greed and Job Performance

Although we use the concept of greed often in everyday life, there is a lack of a consistent definition of greed (Wang et al., 2011). Some definitions emphasize the “cost to others,” which indicates that greed is socially harmful (Balot, 2001; Mussel et al., 2015). For example, (Veselka et al., 2014, p. 76) defined greed as “the tendency to manipulate and betray others for personal gain.” Contrary to the assertion of Balot (2001), Veselka et al. (2014), and Seuntjens et al. (2015) suggested that greed does not necessarily lead to a “cost to others.” They performed a series of prototype analyses to explore lay conceptualizations of greed and found two central elements of greed: “always wanting more” and “never being satisfied” (Seuntjens et al., 2015). As a result, they constructed a working definition of greed as “the experience of desiring to acquire more and the dissatisfaction of never having enough” (Seuntjens et al., 2015, p. 518) and developed the Dispositional Greed Scale to measure greed (Seuntjens et al., 2015). In the present study, we agreed that greed is not intrinsically moral or immoral and thus adopted Seuntjens et al. (2015) definition of greed.

To explore the relationship between greed and performance, we used a two-dimensional performance categorization. Although models of job performance contain many different dimensions, two major categories of job performance can be found across models: task performance and contextual performance (Borman and Motowidlo, 1997). Task performance refers to behaviors that “bear a direct relation to the organization’s technical core, either by executing its technical processes or by maintaining and servicing its technical requirements” (Motowidlo and Van Scotter, 1994, p. 476). Contextual performance includes interpersonal behaviors that “support the broader organizational, social, and psychological environment in which the technical core must function” (Motowidlo and Van Scotter, 1994, p. 476). In the following section, we will theoretically develop the complex pathways from greed to these two types of performance and discuss the underlying mechanism that makes both the positive and negative routes possible.

### Greed Is Positively Related to Performance: The Mediating Role of the Need for Social Status

Social status is the degree to which an individual or group is respected by others (Magee and Galinsky, 2008). The need for social status arises from the social system in which we live (Brown et al., 2011) because higher social status enables individuals to obtain more resources and social benefits. Based on a comprehensive review of diverse empirical literature, Anderson et al. (2015) suggested that the need for social status is a fundamental human motive. Seuntjens et al. (2015) prototype analysis suggested that greed involves not only a desire for material goods but also non-material desires, such as for power and status. Moreover, since the main characteristic of greed is to maximize one’s own interests (Seuntjens et al., 2015), and since improving one’s social status is an effective way to acquire what he or she wants, it is reasonable to argue that people with higher levels of greed may have stronger desires for social status. Research on materialism has also provided indirect evidence that materialism is positively related to status-seeking (Flynn et al., 2016). Materialism refers to the importance that individuals attach to worldly possessions (Belk, 1985) and is found to be closely related to greed (Seuntjens et al., 2015) because both constructs emphasize materialistic desires, while greed also includes non-materialistic objects.

The need for social status has a great impact on one’s performance. Individuals generally value higher social status, and group members tend to accord status to employees who are perceived to be highly contributive (Kilduff et al., 2016). Thus, people with a higher need for social status are more likely to work hard to earn others’ respect and obtain a higher status. The extant literature provides evidence supporting the positive association between the need for social status and job performance. Specifically, for task performance, researchers have suggested that people pursue status by showing their ability to promote group outcomes (Anderson and Kilduff, 2009) or their commitment to group success (Willer, 2009). For contextual performance, researchers have found that status-seeking members are more likely to help others (Flynn et al., 2006) and even sacrifice their own interest to do so (Hardy and Van Vugt, 2006).

Combining the preceding arguments, we propose a critical mediating role of the need for social status, such that greed could facilitate job performance by stimulating employees’ propensity to seek social status.

Hypothesis 1a: The need for social status plays a positive mediating role in the relation between greed and task performance.
Hypothesis 1b: The need for social status plays a positive mediating role in the relation between greed and contextual performance.

### Greed Is Negatively Related to Performance: The Mediating Role of Perceived Distributive Justice

Distributive justice addresses whether tangible or intangible rewards and benefits are distributed to employees fairly (Leventhal, 1976). Although justice depends mainly on the regulation and practice of organizations, researchers have suggested that individuals’ perceptions of fairness and their reactions to unfair outcomes might differ due to individual differences in equity sensitivity (Kickul and Lester, 2001).

According to research on equity sensitivity, benevolent individuals accept a reduced allocation and perceive less injustice (Kickul and Lester, 2001). In contrast, people with higher psychological entitlement, which is a sense that one deserves more and is entitled to more than others (Campbell et al., 2004), are more likely to feel that the outcome is unfair (Huseman et al., 1987; Kickul and Lester, 2001). Since “getting more” is emphasized in the concept of psychological entitlement and earlier research has shown that greed is highly correlated with psychological entitlement (Liu et al., 2019), it is reasonable to speculate that greed may share the same feature. Greedy people always want more and are never satisfied. As a result, they tend to believe that what they have been allocated is less than what they deserve, which could then generate a sense of distributive injustice.

The perception of distributive injustice is detrimental to employees’ performance. According to justice theory (Adams, 1965), if employees feel that their organization is fair and they receive enough rewards from their work, they are more likely to work diligently. In contrast, if employees perceive injustice, one possible way for them to restore justice is to decrease their work performance accordingly. Perceptions of unfairness may deliver a risk signal to employees that their organization does not respect their contribution (Greenberg, 2004), and employees might be inclined to protect their own interests, which could lead to poorer performance (Alesina et al., 2004).

Previous studies have confirmed the impact of perceived justice on task performance and contextual performance. For example, researchers have shown that perceived distributive justice is positively related to job performance (Colquitt et al., 2001) and is negatively related to many counterproductive work behaviors, such as theft (Greenberg, 2002), workplace revenge (Tripp et al., 2002), and sabotage (Ambrose et al., 2002). A meta-analysis showed that employees’ organizational citizenship behaviors, a construct closely related to contextual performance, depend largely on their perceptions of distributive justice and procedural justice (Colquitt et al., 2013).

Based on the preceding arguments, we propose a critical mediating role of perceived distributive justice, through which greed reduces employees’ job performance.

Hypothesis 2a: Perceived distributive justice plays a negative mediating role in the relation between greed and task performance.
Hypothesis 2b: Perceived distributive justice plays a negative mediating role in the relation between greed and contextual performance.

## Materials and Methods

### Participants and Procedure

Online survey questionnaires were administered by the primary researchers responsible for this project. The study was reviewed and approved by the Academic Ethics Committee at the first author’s institution before being conducted. All participants completed the survey online. Before the survey began, a brief description of the survey and the participants’ rights and responsibilities was presented. Knowing that their completion of the survey and their participation was completely voluntary, the participants provided their consent to participate by clicking the “I agree” button. Debriefing information was also provided online at the end of the survey.

Participants were recruited from a paid research participation system^1^, which is used by millions of users in China. Four items in the survey were used as attention checks. The first item was presented soon after the participants provided their consent to participate. The participants were asked whether they were going to answer the questions in the survey seriously. For those who responded by “no,” the survey was terminated immediately. For those who checked “yes,” this item served as a promise from the participant that was aimed at increasing participants’ commitment to complete the survey conscientiously. Another three attention check items were positioned randomly in the survey. A sample item is as follows: “To make sure the screen is working well, please choose the second response option for this item.” The correct answer changed for the other two items. These three items were used to detect participants who failed to read items carefully and chose their responses blindly. Participants who failed any one of the items were regarded as unqualified. The criterion for participant screening was entered into the research participation system beforehand. Only those who passed the attention checks remained in the dataset.

Three hundred and fifteen participants comprised the final sample. All participants had jobs and came from various regions in China. Specifically, 137 (43.49%) participants came from East China, 54 (17.14%) came from South China, 37 (11.75%) came from North China, 31 (9.84%) came from Central China, 16 (5.08%) came from Southwest China, 11 (3.49%) came from Northwest China, and 29 (9.21%) came from Northeast China. The sample consisted of 130 males (41.3%). The participants’ ages ranged from 21 to 59 years (*M* = 32.53, *SD* = 6.70), and the participants’ organizational tenure ranged from 1 to 40 years (*M* = 9.15, *SD* = 6.38). For the highest educational degree achieved, the majority of the participants (*n* = 253; 80.32%) had received a university education, 31 (9.84%) people had received a college education, 21 (6.67%) people had received a master’s level or higher education, 8 (2.54%) people had received a high school education, 1 (0.32%) person had received a primary school education and 1 (0.32%) person had received a middle school education. Each participant received 8 Chinese Yuan for their participation. All research data from this study are available at Mendeley Data.

### Measures

Greed was measured by the Chinese version (Liu et al., 2019) of the seven-item Dispositional Greed Scale originally developed by Seuntjens et al. (2015). All items (e.g., “It doesn’t matter how much I have. I’m never completely satisfied”) were rated using a five-point scale (1 = strongly disagree, 5 = strongly agree), with higher scores representing higher levels of greed. Liu et al. (2019) demonstrated that the psychometric properties of the Chinese version of the Dispositional Greed Scale are satisfactory. In the current study, the Cronbach’s alpha of the scale was 0.86.

The need for social status was measured by the eight-item scale developed by Flynn et al. (2006). All items (e.g., “I want my peers to respect me and hold me in high esteem”) were rated using a six-point scale (1 = strongly disagree, 6 = strongly agree), with higher scores representing higher levels of need for social status. In the current study, the scale was translated into Chinese strictly following the back-translation method (Brislin, 1970). The Cronbach’s alpha of the scale was 0.83.

Perceived distributive justice was measured by the Chinese version (Wang, 2009) of the five-item scale originally developed by Niehoff and Moorman (1993). All items (e.g., “I think that my level of pay is fair”) were rated using a five-point scale (1 = strongly disagree, 5 = strongly agree), with higher scores representing higher levels of perceived distributive justice. Wang (2009) demonstrated that the psychometric properties of the Chinese version of the scale are satisfactory. In the current study, the Cronbach alpha of the scale was 0.82.

Employee performance was measured by the Chinese version (Wang and Liao, 2009) of a 17-item scale originally developed by Van Dyne and LePine (1998). The scale includes two dimensions, namely, task performance (four-items; e.g., “I can fulfill the responsibilities specified in my job description”) and contextual performance (13-items; e.g., “I have volunteered to do things for my work group”). All items were rated on a five-point scale (1 = strongly disagree, 5 = strongly agree), with higher scores representing higher levels of performance. Wang and Liao (2009) demonstrated that the psychometric properties of the Chinese version of the scale are satisfactory. In the current study, the Cronbach’s alphas of the task performance and contextual performance subscales were 0.67 and 0.81, respectively.

We included gender (1 = male, 2 = female), age (in years), and educational level (1 = primary school, 2 = middle school, 3 = high school, 4 = college, 5 = university, 6 = master or higher) in the survey as these biographic variables may influence level of job performance and are commonly controlled in organizational researches (Motowidlo and Van Scotter, 1994; Ambrose et al., 2002; Kilduff et al., 2016). Besides, we also measured organizational tenure (in years), because it may have an impact on employees’ perceptions of distributive justice as well as job performance (De Clercq et al., 2018).

### Analysis Strategy

Before testing the hypothesized model, we examined the measurement models first. All variables were modeled as latent factors with their item means as indicators of the latent constructs. Confirmatory factor analyses (CFAs) using maximum likelihood estimation in IBM SPSS AMOS 26 were performed to check the models. Items with loadings less than 0.30 were deleted, and model fit was ascertained using various indices: the composite reliability should be above 0.60 (Fornell and Larcker, 1981), the comparative fit index (CFI) and the Tucker-Lewis index (TLI) should exceed 0.90, the root mean square error of approximation (RMSEA) should be less than 0.08, and the standardized root mean square residual (SRMR) should be less than 0.05 (Byrne, 2012). Specifically, the latent factor greed was modeled with the seven items as indicators; the latent factor need for social status was modeled with the eight items as indicators; the latent factor perceived distributive justice was modeled with the five items as indicators; the latent factor task performance was modeled with the four items as indicators; and the latent factor contextual performance was modeled with the 13-items as indicators. We next checked whether common method variance existed following Podsakoff et al. (2003) method. Finally, we examined the hypothesized model using latent variable path analyses with structural equation modeling (SEM). The mediating role of perceived distributive justice and need for social status was tested using the bootstrap option (5000 bootstrap samples).

## Results

### Descriptive Statistics

Table 1 presents the means, standard deviations, and correlations among variables. As expected, greed was positively correlated with need for social status (*r* = 0.30, *p* < 0.01) and negatively correlated with perception of distributive justice (*r* = −0.16, *p* < 0.01). In addition, both need for social status and perception of distributive justice were positively correlated with task performance (*r*s > 0.31, *ps* < 0.01) and contextual performance (*r*s > 0.45, *ps* < 0.01).

**TABLE 1:** Descriptive statistics and correlations among the study variables (*N* = 315).

	**1**	**2**	**3**	**4**	**5**	**6**	**7**	**8**	**9**
(1) Gender									
(2) Education	0.02	–							
(3) Age	–0.22^∗∗^	–0.07	–						
(4) OT	–0.23^∗∗^	–0.04	0.93^∗∗^	–					
(5) Greed	–0.06	–0.08	–0.01	0.01	(0.86)				
(6) NFSS	0.01	–0.03	–0.01	0.00	0.30^∗∗^	(0.83)			
(7) DJ	0.07	0.02	–0.11	−0.13^∗^	–0.16^∗∗^	0.31^∗∗^	(0.82)		
(8) TP	0.06	0.10	0.13^∗^	0.11	–0.10	0.31^∗∗^	0.32^∗∗^	(0.67)	
(9) CP	−0.11^∗^	0.06	0.05	0.03	0.00	0.45^∗∗^	0.45^∗∗^	0.44^∗∗^	(0.81)
*M*			32.53	9.15	21.35	35.83	19.28	17.49	52.98
*SD*			6.71	6.38	5.71	4.61	3.08	1.68	5.34

*^∗^*p* < 0.05, ^∗∗^*p* < 0.01. Participants’ gender was dummy coded, with male coded as “1” and female coded as “2.” OT, organizational tenure; NFSS, need for social status; DJ, distributive justice; TP, task performance; CP, contextual performance.*

### Measurement Models

First, we tested every measurement model of the study variables. For greed, which was modeled with six indicators after deleting an item (“I can’t imagine having too many things”) because of low loading (0.29 < 0.3), there was an adequate fit to the data (χ^2^ = 33.26, *p* < 0.001, *df* = 9, χ^2^/*df* = 3.70, CFI = 0.97, TLI = 0.96, RMSEA = 0.09, SRMR = 0.03). For need for social status, after specifying a correlation between reverse-scored items, item 2 (“I am not concerned with my status among my peers”) and item 7 (“I don’t care whether others view me with respect and hold me in esteem”), there was an adequate fit to the data (χ^2^ = 86.81, *p* < 0.001, *df* = 19, χ^2^/*df* = 4.57, CFI = 0.91, TLI = 0.87, RMSEA = 0.11, SRMR = 0.07). For perceived distributive justice, there was a satisfactory fit to the data (χ^2^ = 18.07, *p* < 0.001, *df* = 5, χ^2^/*df* = 3.61, CFI = 0.98, TLI = 0.95, RMSEA = 0.09, SRMR = 0.03). For task performance, there was a satisfactory fit to the data (χ^2^ = 1.24, *p* = 0.54, *df* = 2, χ^2^/*df* = 0.62, CFI = 1.00, TLI = 1.01, RMSEA = 0.00, SRMR = 0.01). For contextual performance, after deleting an item (“I communicate my opinions about work issues to others in my group even if my opinion is different and others in the group disagree with me.”) because of low loading (0.24 < 0.3), there was a satisfactory fit to the data (χ^2^ = 142.83, *p* < 0.001, *df* = 65, χ^2^/*df* = 2.20, CFI = 0.90, TLI = 0.88, RMSEA = 0.07, SRMR = 0.05). The factor loadings of all items measuring study variables ranged between 0.40 and 0.75 (Table 2 shows the results).

**TABLE 2:** Item loadings for each construct in the best fitting structural model.

**Variables**	**Item**	**Loadings**	**Composite reliability**
Greed	DG1	0.75	0.89
	DG2	0.72	
	DG3	0.66	
	DG4	0.77	
	DG5	0.80	
	DG6	0.79	
Need for social status	NFSS1	0.47	0.82
	NFSS2	0.44	
	NFSS3	0.73	
	NFSS4	0.75	
	NFSS5	0.62	
	NFSS6	0.72	
	NFSS7	0.48	
	NFSS8	0.62	
Perceived distributive justice	PDJ1	0.63	0.83
	PDJ2	0.75	
	PDJ3	0.72	
	PDJ4	0.75	
	PDJ5	0.64	
Task performance	TP1	0.69	0.66
	TP2	0.47	
	TP3	0.55	
	TP4	0.57	
Contextual performance	CP1	0.53	0.81
	CP2	0.40	
	CP3	0.49	
	CP4	0.42	
	CP5	0.50	
	CP6	0.46	
	CP7	0.48	
	CP8	0.55	
	CP9	0.60	
	CP11	0.58	
	CP12	0.49	
	CP13	0.63	

To ensure that common method variance was not a problem, we tested an alternative model with all factors collapsed into one latent factor (Podsakoff et al., 2003; Akkermans et al., 2018). This model showed a significantly worse fit than the final measurement model (Δχ^2^ = 2093.93, Δ*df* = 4, *p* < 0.001). Hence, our hypothesized measurement model showed a better fit to the data.

### Structural Model

We finally tested a hypothesized structural model controlling the effects of demographic variables on task performance and contextual performance. With a correlation between age and organizational tenure, fit indices of the overall hypothesized structural model showed that the model fit the data adequately (χ^2^ = 1198.70, *p* < 0.001, *df* = 727, χ^2^/*df* = 1.65, CFI = 0.90, TLI = 0.89, RMSEA = 0.05, SRMR = 0.07).

**FIGURE 1:**
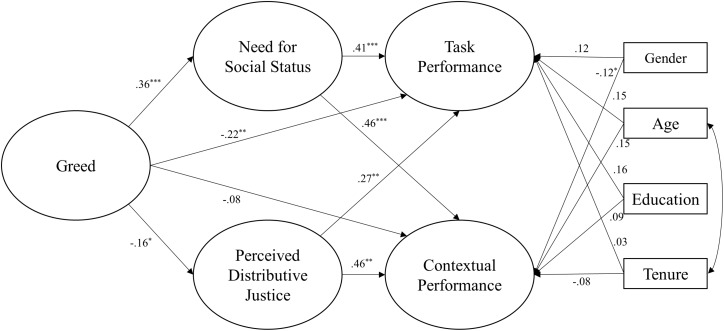
Results of the empirical model test. ^∗^*p* < 0.05, ^∗∗^*p* < 0.01, ^∗∗∗^*p* < 0.001.

As Figure 1 suggests, greed had a significantly negative direct effect on task performance (*B* = −0.09, SE = 0.03, β = −0.22, *p* = 0.007). The direct effect of greed on contextual performance was non-significant (*B* = −0.02, SE = 0.02, β = −0.08, *p* = 0.206).

Next, greed was positively related to need for social status (*B* = 0.15, SE = 0.03, β = 0.36, *p* < 0.001), and need for social status was positively related to both task performance (*B* = 0.43, SE = 0.10, β = 0.41, *p* < 0.001) and contextual performance (*B* = 0.34, SE = 0.07, β = 0.48, *p* < 0.001). In addition, need for social status was a significant mediator of the relations between greed and task performance [standardized indirect effect = 0.06, *p* < 0.001, 95% CI = (0.03, 0.12)] and a significant mediator of the relations between greed and contextual performance [standardized indirect effect = 0.05, *p* < 0.001, 95% CI = (0.03, 0.08)]. The results support both Hypotheses 1a and 1b.

Finally, greed was negatively related to perceived distributive justice (*B* = −0.09, SE = 0.04, β = −0.16, *p* = 0.03), and perceived distributive justice was positively related to both task performance (*B* = 0.21, SE = 0.06, β = 0.30, *p* = 0.005) and contextual performance (*B* = 0.25, SE = 0.04, β = 0.46, *p* = 0.001). In addition, perceived distributive justice was a significant mediator of the relations between greed and task performance [standardized indirect effect = −0.02, *p* = 0.02, 95% CI = (−0.05, −0.00)] and a significant mediator of the relations between greed and contextual performance [standardized indirect effect = −0.02, *p* = 0.02, 95% CI = (−0.05, −0.00)]. The results support both Hypotheses 2a and 2b.

## Discussion

Greed has attracted increasing research interest in recent years. Researchers have explored how greed is related to one’s personality (e.g., Gilliland and Anderson, 2011), values and beliefs (e.g., Levine, 2005; Liu et al., 2019), and ethical behaviors (e.g., Seuntjens et al., 2019). The current study is one of the first empirical investigations of the dual effects of greed on employees’ performance and the underlying mechanisms. The results confirmed the hypotheses that greed has opposite effects on one’s performance. Specifically, greed could motivate individuals to work hard but also could diminish their desire to demonstrate good performance. In addition, we found that greed promoted performance through the intermediary effect of the need for social status but simultaneously inhibited performance through perceived distributive justice. The pattern of the relationship generally held for both task and contextual performance.

The two key elements of greed are “wanting more” and “never being satisfied with what one already has” (Seuntjens et al., 2019). In organizational settings, these features have a very large influence on employees’ attitudes toward valuable material resources (such as money) and non-material resources (such as power). The dual effect of greed on people’s performance in organizations and the mediating role of need for social status and perceived distributive justice could all be traced back to the key features of greed.

In terms of the “wanting more” aspect of greed, a higher social status is necessary given that resources are generally distributed according to one’s standing in the social hierarchy of the organization (Highhouse et al., 2016). The findings of the current study provide some empirical evidence that the greedier an individual is, the stronger his or her need for social status, and the higher his or her level of performance. These results are also consistent with previous research findings that greedy individuals are more productivity-oriented and have a stronger desire to win (Krekels and Pandelaere, 2015). Although some researchers have claimed that greed is socially harmful (Krekels et al., 2011), greedy people’s need for higher social status may lead to beneficial behaviors toward both themselves (task performance) and others (contextual performance), which ultimately benefits organizations.

The “dissatisfaction” aspect of greed inevitably influences people’s attitudes toward what they have already been allocated. In other words, the dissatisfaction experienced by greedy people casts doubt on the distributive justice of organizations. As a result, the subjective perception of distributive injustice is likely to undermine employees’ performance. Although the effect size is small, the findings of the current study provide some empirical evidence that the greedier an individual is, the lower his or her perceived distributive justice and the poorer his or her level of performance. The results of the current study contribute to the literature on equity sensitivity (Huseman et al., 1987) by suggesting that greedy people, similar to those people with higher psychological entitlement, are hypersensitive to distributive justice.

Based on the above findings, greed is likely to be a double-edged sword for employees’ job performance in organizations. Differing attitudes toward greed remain in the literature. As mentioned previously, economists tend to affirm greed’s positive impacts (Greenfeld, 2003), while most psychological studies have focused on its negative effects (Smith, 2003; Cohen et al., 2009; Seuntjens et al., 2019). Although some researchers have argued the dual effects of greed, they often have developed their propositions from an interpersonal perspective, i.e., that greed could benefit the individual himself or herself but do harm to others (Bruhn and Lowrey, 2012). This idea is consistent with Hume (2001) claim that greed, on the one hand, encourages people to do better and, on the other hand, has devastating consequences for society.

The current study extends the double-edged nature of greed to the intrapersonal domain. We found that even for greedy people themselves, greed could simultaneously facilitate and impede their performance. These findings are important contributions to the field because they suggest that greed is not necessarily good or bad. Its valence depends on what is motivating greedy people. If the desire for social status is stimulated, greedy people could contribute to organizations by improving not only their task performance but also their contextual performance. However, if greedy people are haunted by the perceived distributive injustice of an organization, their performance might be negatively affected. For organizations, the conditions under which greed could generate beneficial outcomes is a more meaningful question than whether greed is good or bad or should be encouraged or curbed. The important practical implication of the current study is that organizations should both address greedy employees’ social status concerns and ensure that they are treated fairly so that they can fully utilize the talents of greedy people and channel their energy in a more constructive direction.

### Limitations and Future Directions

First, given that the current study employed a cross-sectional design, further longitudinal study is needed before any causal relationships can be established. For example, the current study proposed that greed is a determinant of the need for social status. However, it is also possible that the need for social status might make people greedy. A cross-lagged panel design would help researchers to confirm the causality relations in our model.

Second, the current study used self-rating scales to measure employees’ task and contextual performance as well as other constructs, so common method variance may be an issue. However, following Podsakoff et al.’s (2003) method, we found that common method variance was not a serious problem in the current study. Meanwhile, researchers have found that self-rated performance is highly positively related to peer-rated performance (Demerouti et al., 2014). Moreover, self-rated performance has a unique advantage in our study because supervisors’ ratings may be influenced by impression management behaviors (De Cuyper et al., 2014). Future studies could use multisource data, including self-rating scales, evaluations provided by employees’ supervisors, and objective performance indexes, to further test the proposed model in the current study.

Third, future studies could explore the boundary condition of this two-pathway model. The current study found that both mediators existed between greed and job performance, and it is reasonable to speculate that some key contextual factors may influence which path plays a more important role. For instance, Knight and Mehta (2017) found a joint effect of social status and hierarchy stability on performance. Specifically, higher status individuals performed better than lower status individuals when hierarchy stability was high. This finding suggests that the effect of the need for social status may exist only when hierarchy stability is low, under which circumstance people with low status have more access to higher status.

Finally, the generalizability of the current results could be examined in different cultures. The cultural context of the current study, China, emphasizes social status much more than many other countries (Xi, 2016). Although the desire for status is fundamental and universal (Anderson et al., 2015), the importance people attach to it and the acceptable methods that people use to acquire it may vary among cultures. Huberman et al. (2004) compared the intrinsic value of status in the United States, Germany, Finland, Turkey, and China. They found that Chinese people pay more attention to social status and show more status-seeking behaviors than people from the other countries (Huberman et al., 2004). In addition, traditional Chinese values encourage people to improve their social status through their efforts and to pursue their wealth and status in moral ways (Xi, 2016). Future studies in different cultures are necessary to provide supporting evidence for the dual effect model of greed.

## Data Availability

The datasets generated for this study are available on request to the corresponding author.

## Ethics Statement

This study was carried out in accordance with the recommendations of the American Psychological Association Ethics Guidelines and the Committee on Human Protection and Ethics in Psychology. All subjects gave written informed consent in accordance with the Declaration of Helsinki. The protocol was approved by the Committee on Human Protection and Ethics in Psychology at Beijing Normal University.

## Author Contributions

YZ developed the study concept, and performed the data analysis and interpretation. YZ and XS created the study design and drafted the manuscript. SL performed the testing and data collection. GX provided the revisions. All authors approved the final version of the manuscript for submission.

## Conflict of Interest Statement

The authors declare that the research was conducted in the absence of any commercial or financial relationships that could be construed as a potential conflict of interest.

## Footnotes

1 www.sojump.com
